# The Protective Effect of Bee Venom on Fibrosis Causing Inflammatory Diseases

**DOI:** 10.3390/toxins7114758

**Published:** 2015-11-16

**Authors:** Woo-Ram Lee, Sok Cheon Pak, Kwan-Kyu Park

**Affiliations:** 1 Department of Pathology, College of Medicine, Catholic University of Daegu, 3056-6, Daemyung-4-Dong, Nam-gu, Daegu 705-718, Korea; E-Mail: woolamee@cu.ac.kr; 2 School of Biomedical Sciences, Charles Sturt University, Panorama Avenue, Bathurst, NSW 2795, Australia; E-Mail: spak@csu.edu.au

**Keywords:** bee venom, inflammation, liver fibrosis, atherosclerosis, skin disease

## Abstract

Bee venom therapy is a treatment modality that may be thousands of years old and involves the application of live bee stings to the patient’s skin or, in more recent years, the injection of bee venom into the skin with a hypodermic needle. Studies have proven the effectiveness of bee venom in treating pathological conditions such as arthritis, pain and cancerous tumors. However, there has not been sufficient review to fully elucidate the cellular mechanisms of the anti-inflammatory effects of bee venom and its components. In this respect, the present study reviews current understanding of the mechanisms of the anti-inflammatory properties of bee venom and its components in the treatment of liver fibrosis, atherosclerosis and skin disease.

## 1. Introduction

Bee venom is a natural toxin produced by the honey bee and it has a prime role of defense for the bee colony [1,2,3]. It has an efficient and complex mixture of substances designed to protect bees against a broad diversity of predators [2]. Bee venom possesses various peptides including melittin, apamin, adolapamin and mast cell degranulating peptide [4,5]. It also contains enzymes, biologically activity amines and non-peptide components. Enzymes are composed of phospholipase A2 (PLA2), hyaluronidase, acid phosphomonesterase, α-d-glucosidase and lysophospholipase, as well as non-peptides such as histamine, dopamine and norepinephrine [6]. Bee venom therapy is a treatment modality that may be thousands of years old and involves the application of live bee stings to the patient’s skin or, in more recent years, the injection of bee venom into the skin with a hypodermic needle [7]. Many experiments on the biological and pharmacological activities of bee venom have been carried out [2,3,4,5,6,7,8]. Majority of these studies have proven the effectiveness of bee venom in treating pathological conditions such as arthritis [9], pain [10,11] and cancerous tumors [12,13] among others.

The major component of bee venom is melittin, which comprises approximately 50% of the dry weight of bee venom. It is a small linear peptide composed of 26 amino acid residues [14]. Melittin has multiple effects, including anti-bacterial, anti-viral and anti-inflammatory, in various cell types [15,16]. Recent studies have shown that melittin can induce cell cycle arrest, cell growth inhibition and apoptosis in various tumor cells [17,18,19]. When several melittin peptides accumulate in the cell membrane, phospholipid packing is severely disrupted, thus leading to cell lysis [16]. Melittin triggers not only the lysis of a wide range of plasmatic membranes but also of intracellular ones such as those found in mitochondria. PLA2 and melittin act synergistically, breaking up membranes of susceptible cells and enhancing their cytotoxic effect [20]. However, other paper reported that melittin at concentrations below 2 μM does not disrupt cell membranes of leukocytes [21]. In addition, another paper reported that an optimal dose of melittin protects TGF-β1-induced apoptotic activation of hepatocytes by inhibiting the activation of the Bcl-2 family of proteins, caspases and poly (adenosine diphosphate-ribose) polymerase (PARP) cleavage [22].

Apamin is an integral part of bee venom, accounting for about 2%–3% of its dry weight [3]. It is a peptide neurotoxin comprising 18 amino acid residues that is tightly cross-linked by the presence of two disulphide bonds [5]. Apamin is well known for its pharmacological property of irreversibly blocking Ca^2+^-activated K^+^ (SK) channels [23]. These channels link intracellular calcium transients to changes of the membrane potential by promoting K^+^ efflux following increases of intracellular calcium during an action potential [24]. In a previous paper, it was reported that apamin inhibited pro-inflammatory cytokines in lipopolysaccharide (LPS) with fat diet-induced atherosclerotic animal model [25]. Furthermore, a recent study has examined its biological and pharmacological activities [26]. However, little is known about the molecular mechanisms and the levels of gene regulation involved in the anti-inflammatory process. As such, this review focuses on overview of recent research on anti-inflammatory properties of bee venom and its components in liver fibrosis, atherosclerosis and inflammatory skin disease. Moreover, we review possible mechanisms of bee venom for alleviating or preventing the inflammatory diseases.

## 2. Anti-Inflammatory Effect of Bee Venom on Liver Fibrosis

Liver fibrosis occurs with chronic hepatic damage in a variety of liver diseases including viral hepatitis, alcoholic hepatitis and primary sclerosing cholangitis [27]. In these conditions, fibrotic liver shows changes in tissue architecture and extracellular matrix composition that ultimately compromise organ function [28,29,30,31]. The processes of liver repair and of fibrogenesis resemble that of a wound-healing process. Viral infection, alcoholic or drug toxicity, or any other factors that cause damage to hepatocytes, elicit an inflammatory reaction in the liver [14]. Following injury, an acute inflammation response takes place resulting in moderate cell necrosis and extracellular matrix damage [32]. Chronic ethanol consumption is associated with serious and potentially fatal alcohol-related liver diseases such as fatty liver, alcoholic hepatitis and cirrhosis [33]. It is currently understood that the pathogenesis of these diseases is related to apoptosis [34]. Pro-inflammatory cytokine, TNF-α can induce multiple mechanisms that initiate apoptosis in hepatocytes, which leads to liver injury [35]. A paper reported that an optimal dose of bee venom exerts anti-apoptotic effects against ethanol-induced injury to hepatocytes via the mitochondrial pathway [34]. Thus, bee venom protects hepatocyte against TNF-α with actinomycin D induced apoptosis. Low concentrations of bee venom resulted in anti-apoptotic effects that were associated with a decrease in the level of proteolytic fragments of caspases and PARP [36]. Furthermore, a recent study indicates that bee venom inhibits CCL_4_-induced hepatic fibrosis through suppression of fibrogenic cytokines in liver fibrosis animal model. This study shows that bee venom down-regulated pro-inflammatory cytokines such as TNF-α and IL-1β. It has been demonstrated that collagen gene expression is regulated by TNF-α at a transcription level and IL-1β exerts a stimulatory effect on the synthesis of extracellular matrix components [37]. Transforming growth factor (TGF)-β is a multifunctional cytokine that mediates cellular differentiation, growth and apoptosis [38]. Park *et al.* reported that TGF-β1 decreased cell viabilities and induced hepatocyte apoptosis. However, adding the 10 ng/mL of bee venom significantly increased the viability of TGF-β1-treated hepatocyte [39]. In addition, Lee and colleagues demonstrated that an optimal dose of melittin exerts anti-apoptotic effects against TGF-β1-induced injury to hepatocytes via the mitochondrial pathway [22]. As such, these papers found that an optimal dose of bee venom and melittin can serve to protect cells against TGF-β1-mediated injury.

The nuclear transcription factor NF-κB is the key player in the development of chronic inflammatory diseases [40]. This transcription factor-involved-pathway is one of the main signaling pathways activated in response to pro-inflammatory cytokines. In addition, activation of this pathway plays a central role in inflammation through the regulation of genes encoding various growth factors [41]. Park *et al.* suggested that melittin attenuates liver injury in thioacetamide-treated mice through modulating inflammation and fibrogenesis [14]. These authors investigated the mechanism for suppression of NF-κB transcription by melittin in TNF-α-treated hepatocytes, examining the effect of melittin on NF-κB promoter activity by transiently transfected luciferase reporter plasmid containing the NF-κB promoter sequence. Melittin significantly inhibited NF-κB promoter activity and NF-κB DNA binding activity in TNF-α-treated hepatocytes. These results suggest that melittin suppresses NF-κB activation, leading to an inhibition of hepatocyte apoptosis [42].

Hepatic stellate cells (HSCs) are perisinusoidal cells residing in the space of Disse. During injury, in response to inflammatory and other stimuli, these cells adopt a myofibroblast-like phenotype and represent the cornerstone of the fibrotic response in the liver [42,43]. Once activated, HSCs up-regulate gene expression of extracellular matrix (ECM) components, matrix-degrading enzymes and their respective inhibitors, resulting in matrix remodeling and accumulation at sites with abundant activated HSCs [31,44]. Park *et al.* reported that melittin inhibited TNF-α secretion in the TNF-α-treated HSCs. Furthermore, melittin inhibited the TNF-α-induced expression of IL-1β and IL-6, especially with 0.5 mg/mL of melittin. This article also showed that melittin protected against thioacetamide-induced liver fibrosis by suppressing liver inflammation and fibrogenesis through the NF-κB signaling pathway. In addition, its anti-fibrotic effect may be attributed to modulation of the inflammatory effect in the activated HSC [14].

Acute hepatic failure is characterized by hepatic encephalopathy, severe coagulopathy, jaundice and hydroperitoneum [45,46]. Administration of a subtoxic dose of D-galactosamine together with LPS has often been used for preparing an animal model with endotoxemic shock and acute liver failure [47]. Upon stimulation with D-galactosamine and LPS, secretion of various pro-inflammatory cytokines and hepatic necrosis occur, which leads to the decreased levels of antioxidant enzymes [48,49]. This liver injury has been associated with significant increases in alanine aminotransferase (ALT) activity and TNF-α level in serum, ultimately leading to extremely high lethality [50]. Park and co-investigators found that melittin prevents D-galactosamine/LPS-induced liver failure by suppressing apoptosis and the inflammatory response in the mouse liver [51]. Melittin decreased the high rate of lethality, alleviated hepatic pathological injury, attenuated hepatic inflammatory responses and inhibited hepatocyte apoptosis. This study provides evidence that melittin may offer an alternative for the prevention of acute hepatic failure. 

Some evidence suggests that adult hepatocytes play a role by way of epithelial mesenchymal transition (EMT) in the accumulation of activated fibroblasts [52,53]. EMT is a dynamic cellular program in which polarized epithelial cells lose epithelial properties, undergo morphological changes and acquire mesenchymal characteristics [54]. Hepatocytes can transdifferentiate into mesenchymal cells by EMT and deposit collagen in the liver during chronic injury [55]. A recent study has investigated the anti-fibrosis or anti-EMT mechanism by examining the effect of apamin on TGF-β1-treated hepatocytes or CCl_4_-injected animal model. This article demonstrated that administration of apamin significantly increased the expression of epithelial marker E-cadherin and decreased mesenchymal marker vimentin in the TGF-β1-treated hepatocytes. In particular, apamin suppressed the expression of Smad-independent and Smad-dependent signaling pathways in hepatocytes. These results demonstrate the potential of apamin for the prevention of EMT progression induced by TGF-β1 *in vitro* [26].

PLA2 from bee venom is a prototypic group III enzyme that hydrolyzes fatty acids and it has been reported that melittin in bee venom enhances the activity of PLA2 [56,57]. In addition, it has been shown that PLA2 prevents neuronal cell death and spinal cord injury [58,59]. Kim *et al.* demonstrated that PLA2 protects against hepatic dysfunction and induces anti-inflammatory cytokine production in acetaminophen-injected mice. This study suggests that PLA2 may have therapeutic potential in preventing acetaminophen-induced hepatotoxicity [60].

## 3. Anti-Inflammatory Effect of Bee Venom on Atherosclerosis

Atherosclerosis is a chronic inflammatory disease of the arteries resulting from interactions among lipids, monocytes and arterial wall cells [61]. The early stage of atherosclerosis involves the activation of the vascular endothelium in response to many stimuli, such as low-density lipoproteins, free radicals, infectious microorganisms, shear stress, hypertension and toxins from smoking [62]. In the progression of atherosclerosis, the proliferation and migration of vascular smooth muscle cell (VSMC) play an important role in causing stenosis or intimal thickening [63]. The migration and proliferation of VSMC is caused by pathological phenomena such as the accumulation of inflammatory cells and the release of pro-inflammatory cytokines [64,65]. In addition, abundance of macrophages is observed in atherosclerotic lesions, and early lesions of atherosclerosis are characterized by the infiltration of monocyte/macrophages and the presence of macrophage foam cells [63]. Macrophages are multi-potent inflammatory cells with the capacity for synthesis and secretion of pro-inflammatory cytokines such as TNF-α, IL-1β, IL-8 and IL-6 [61,66]. Particularly, TNF-α is reportedly involved in the development of early atherosclerosis by up-regulating vessel wall chemokine and expression of adhesion molecules such as intercellular adhesion molecule (ICAM)-1 and vascular cell adhesion molecule (VCAM)-1 in the aorta [67,68]. The up-regulation of the endothelial adhesion molecules promotes the development of atherosclerotic lesions in rabbits [69], subhuman primates [70] and humans [71,72]. Therefore, the suppression of cell adhesion molecule expression and macrophage accumulation at the level of the endothelium is of particular significance with respect to the management of the vascular inflammatory process. Some of study demonstrated that bee venom inhibits the development of atherosclerosis in C57BL/6 mice induced by injected LPS with feeding of an atherogenic diet. This is likely due to mechanisms involving anti-hypertriglyceridemic and anti-inflammatory effects of bee venom [73]. This study suggested that reduction of adhesion molecules and inflammatory factors by bee venom may be a protection against the atherosclerotic lesion formation.

The increased potential for growth of VSMC is a key abnormality in the development of atherosclerotic lesions [63]. It is well known that, in response to a platelet-derived growth factor (PDGF), VSMC can initiate highly conserved signaling events, which lead to either cell migration or proliferation [74]. Given the nature of VSMC in atherosclerosis, its apoptosis is beneficial in that it offers protection to the walls of arteries against proliferative restenosis induced by arterial injury, including arterial balloon angioplasty or stent implantation [75,76,77,78,79,80]. Son *et al.* reported that the anti-atherosclerotic effects of melittin were identified by interfering with the induction of apoptosis, inhibiting the proliferation of aortic VSMC and inhibiting downstream molecules of the PDGF receptor [8]. In addition, several studies have investigated the role of type IV collagaenase or gelatinase (MMP-2 and 9) in the regulation of VSMC behavior both *in vitro* and *in vivo* [81,82]. MMP-9 is expressed in the initial stage of the smooth muscle cell (SMC) migration, whereas the MMP-2 activity is observed at a later stage after arterial injury [83]. The synthesis and secretion of MMP-9 can be stimulated by various stimuli, including TNF-α and PDGF, during pathological processes such as atherosclerosis and inflammation [82,84,85]. Jeong *et al.* investigated the effects of melittin on TNF-α-induced migration of human aortic SMCs. The study found that melittin suppresses TNF-α-induced MMP-9 expression by inhibiting its gene transcription, but not by regulating the tissue inhibitor or metalloproteinases. Additionally, suppression of the human aortic SMC migration by melittin appeared to block the MMP-9 expression by inhibiting the NF-κB signal pathway. This study suggested that melittin is a potential agent for the prevention of vascular disorders related to the VSMC migration [65]. Recently, numerous basic research studies have indicated that TNF-α accelerates atherosclerosis in mice. Moreover, IL-1β, which plays an important role in the mediation of inflammatory responses and in the pathogenesis of atherosclerosis, is secreted by macrophages in atherosclerotic lesions [86,87]. Kim *et al.* investigated the protective effects of melittin on serum lipid profiles, pro-inflammatory cytokines, pro-atherosclerotic proteins and adhesion molecule levels in an LPS/high fat-induced mouse model of atherosclerosis and monocyte-derived macrophages. The major finding is that melittin inhibits LPS/high fat-induced expression levels of inflammatory cytokines and adhesion molecules such as TNF-α, IL-1β, ICAM and VCAM. Furthermore, the mechanisms are partly attributable to the inhibition of the NF-κB signaling pathway in LPS-treated monocyte-derived macrophages [88].

Several studies have confirmed that some calcium channel blockers can decrease the area of atherosclerotic lesions, production of oxidative stress and expression of inflammatory cytokines without conspicuously affecting blood lipid levels [89]. Kim *et al.* valuated the anti-atherosclerotic or anti-apoptotic mechanisms of apamin in THP-1-derived macrophages. Treatment of cells with oxLDL significantly promoted the accumulation of lipids and expression of apoptotic proteins. However, treatment of macrophages with apamin inhibited apoptosis through the regulation of Bcl-2 family, caspase-3 and PARP apoptotic pathway. *In vivo*, apamin attenuated apoptotic cell death in atherosclerotic mice [90]. These authors also investigated the protective effect of apamin on LPS/fat-induced atherosclerotic mice. The treated mice showed a large number of atherosclerotic lesions in the aorta. However, treatment with apamin predominantly attenuated atherosclerotic lesions, lipid, Ca^2+^ levels, pro-inflammatory cytokines, adhesion molecules, fibrotic factors and macrophage infiltrations. In regard to mechanism, it was found that treatment with apamin in THP-1-derived macrophages suppresses inflammatory responses by a decrease of the NF-κB signal pathway. Therefore, this study suggests that apamin plays an important role in monocyte/macrophage inflammatory processing and may be of potential value for preventing atherosclerosis [25].

The proliferation of VSMC is governed by the cell cycle, a common convergent point for proliferative signaling cascades [91]. The cell cycle, which consists of three distinct sequential phages (G0/G1, S and G2/M), regulates cellular proliferation [92]. Generally, the cell cycle is tightly regulated by the activity of cycle-dependent kinase (CDK) and the specific regulatory cyclin complex. Specific CDKs are sequentially activated during different phases of the cell cycle [93]. A recent study examined the cellular mechanisms by which apamin inhibits cell cycle progression of the cells exposed to PDGF. This study also investigated the inhibitory effect of apamin on PDGF-induced VSMC proliferation and migration. The results showed that PDGF-treated-VSMC was decreased in cell proliferation and migration through the regulation of cyclin D1, CDK 4, cyclin E and CDK 2. Notably, 2 μg/mL of apamin inhibited the PDGF stimulated proliferation of VSMC through blocking PDGF signaling pathway [94].

## 4. Anti-Inflammatory Effect of Bee Venom on Skin Disease

Acne vulgaris is the most common skin disease of the pilosebaceous follicle and results in non-inflammatory and inflammatory lesions [95]. *Propionibacterium acnes* (*P. acnes*) is a major contributing factor to the inflammatory component of acne [96]. *P. acnes* contributes to the inflammatory reaction of acne by inducing monocytes and keratinocytes to produce pro-inflammatory cytokines, including IL-1β, IL-8 and TNF-α [97]. The induction of these cytokines by *P. acnes* is mediated by Toll-like receptor (TLR) 2 [98]. Various therapeutic agents, including antibiotics for acne, have been used to inhibit inflammation or bacteria growth. However, antibiotics may lead to the emergence of resistant pathogens and side effects [99]. Thus, research recently focused on the anti-inflammatory property of bee venom. This included the effect of heat-killed *P. acnes* on human keratinocyte and monocyte cell lines. Kim *et al.* investigated the anti-inflammatory effects of bee venom in heat-killed *P. acnes*-treated HaCaT and THP-1 cells, as revealed by ELISA analysis and Western blotting by measuring the pro-inflammatory cytokines and chemokines. Heat-killed *P. acnes* markedly increased the secretion of TNF-α, IL-8 and IFN-γ in HaCaT and THP-1 cells. However, bee venom treatment decreased the secretion of those cytokines. In addition, bee venom inhibited heat-killed *P. acnes*-induced TLR2 expression in HaCaT cells. These results suggest that bee venom blocked TLR2 expression and suppressed the production of pro-inflammatory cytokines induced by *P. acnes* in HaCaT and THP-1 cells [100]. Another recent study conducted by An *et al.* reported that bee venom has a potential anti-bacterial effect against inflammatory skin disease. In this context, *P. acnes* was intradermally injected into ears of ICR mice. Following the injection, bee venom mixed with vaseline was applied to the skin surface of the ear. Histological observation revealed that the *P. acnes* injection induced a considerable increase in the number of infiltrated inflammatory cells and inflammatory cytokines. By contrast, the bee venom treated ears showed noticeably reduced ear thickness. Additionally, bee venom significantly inhibited the number of TNF-α and IL-1β positive cells [101]. Han *et al.* investigated the biological effect of bee venom treatment on keratinocyte migration *in vitro*. Migration assays showed that the distance of cell migration was dramatically increased in the experimental cells exposed to bee venom. This finding suggests that human epidermal keratinocyte migration occurred more rapidly in the bee venom treated cell, indicating that bee venom stimulates keratinocyte migration. Therefore, bee venom could be applied topically to accelerate wound healing by cell regeneration process [6].

During an inflammatory response, TLR activation results in the activation of the MAPK and the transcription factor NF-κB signaling pathways. These pathways then modulate inflammatory gene expression, which is crucial in shaping the innate immune response within the inflammatory skin disease [102]. Lee *et al.* investigated the effects of melittin in the production of inflammatory cytokines in heat-killed *P. acnes*-treated HaCaT cells. Furthermore, the molecular pathogenesis of anti-inflammatory effects of melittin was investigated in living *P. acnes*-induced inflammatory skin disease animal model. Administration of heat-killed *P. acnes* increased expression of IKK, IκB and NF-κB in HaCaT cells. However, the addition of melittin reduced IKK, IκB and NF-κB phosphorylation. These results indicate that treatment with melittin abrogated the effect of *P. acnes* in altering the expression through NF-κB signaling. The same study investigated whether melittin modulates MAPK signaling in heat-killed *P. acnes*-treated HaCaT cells. Findings showed that phosphorylated p38 was markedly increased after treatment with heat-killed *P. acnes*; however, phosphorylated p38 was decreased after treatment with melittin. These results underscore the theory that melittin inhibits pro-inflammatory cytokine expression by suppression of p38 MAPK phosphorylation in heat-killed *P. acnes*-treated HaCaT cells [103].

## 5. Conclusions

Due to the rising prevalence of side effects from pharmacological approach to inflammatory disease, there is a pressing need for better treatment to alleviate the symptoms of these disorders. The present review is the first to focus on how bee venom and its major components may be incorporated into therapy for inflammatory diseases. We propose that bee venom may serve as an inflammation modulator that subsequently affects the liver fibrosis, atherosclerosis and skin disease. Bee venom and its components regulate pro-inflammatory cytokines in hepatocyte and liver fibrosis animal model. In the atherosclerosis animal model, bee venom appears to inhibit the inflammatory reactions and VSMC proliferation. Furthermore, bee venom seems to accelerate wound healing and antibacterial therapy for the treatment of inflammatory skin disease through the regulation of inflammatory signaling pathway. Collectively, therapy using bee venom and its major components is considered a useful clinical approach for the treatment of inflammatory diseases. In addition, further studies including experimental elucidation of optimal dose, allergic reaction and side effects will lead to a potential therapeutic alternative for inflammatory disease. Since bee venom contains a number of other components, advances in modern sequencing techniques will provide an arsenal of new possibilities to combat other inflammation related diseases.
